# The environmental hypersensitivity symptom inventory: metric properties and normative data from a population-based study

**DOI:** 10.1186/0778-7367-71-18

**Published:** 2013-07-09

**Authors:** Steven Nordin, Eva Palmquist, Anna-Sara Claeson, Berndt Stenberg

**Affiliations:** 1 Department of Psychology, Umeå University, Umeå SE-90187, Sweden; 2 Department of Public Health and Clinical Medicine, Umeå University, Umeå 414 SE-90187, Sweden

**Keywords:** Chemical intolerance, Electromagnetic fields, Hyperacusis, Idiopathic environmental intolerance, Prevalence, Sick building syndrome

## Abstract

**Background:**

High concomitant intolerance attributed to odorous/pungent chemicals, certain buildings, electromagnetic fields (EMF), and everyday sounds calls for a questionnaire instrument that can assess symptom prevalence in various environmental intolerances. The Environmental Hypersensitivity Symptom Inventory (EHSI) was therefore developed and metrically evaluated, and normative data were established. The EHSI consists of 34 symptom items, requires limited time to respond to, and provides a detailed and broad description of the individual’s symptomology.

**Methods:**

Data from 3406 individuals who took part in the Västerbotten Environmental Health Study were used. The participants constitute a random sample of inhabitants in the county of Västerbotten in Sweden, aged 18 to 79 years, stratified for age and gender.

**Results:**

Exploratory factor analysis identified five significant factors: airway symptoms (9 items; Kuder-Richardson Formula 20 coefficient, KR-20, of internal consistency = 0.74), skin and eye symptoms (6 items; KR-20 = 0.60), cardiac, dizziness and nausea symptoms (4 items; KR-20 = 0.55), head-related and gastrointestinal symptoms (5 items; KR-20 = 0.55), and cognitive and affective symptoms (10 items; KR-20 = 0.80). The KR-20 was 0.85 for the entire 34-item EHSI. Symptom prevalence rates in percentage for having the specific symptoms every week over the preceding three months constitute normative data.

**Conclusions:**

The EHSI can be recommended for assessment of symptom prevalence in various types of environmental hypersensitivity, and with the advantage of comparing prevalence rates with normality.

## Background

Health symptoms attributed to environmental agents are an extensive occupational and public health problem. Apart from toxic and allergenic substances, symptoms are commonly attributed to chemicals and biological materials (e.g., mold) that generate odor and sensory irritation (e.g., pungency), to electrical equipment that generate electromagnetic fields (EMF), and to mechanical phenomena that generate sound. Health effects of exposure to strong EMF are well documented, and such exposure is controlled by regulations and guidelines [1]. However, there is no existing evidence for health effects from low-level EMF exposure. Instead there is evidence for a nocebo effect in triggering acute health effects [2-4]. Nevertheless, health problems evoked in the presence of electrical equipment is a concern.

Clinical diagnoses for these environmental intolerances (EI) include multiple chemical sensitivity (MCS) [5], nonspecific building-related symptoms (sick building syndrome) [6], idiopathic environmental intolerance attributed to electromagnetic fields (IEI-EMF) [7], and sound sensitivity (hyperacusis) [8]. As many as 6.3% of a general Swedish population report having a physician-based diagnosis of at least one of these four intolerances, and 21.6% report an intolerance (not necessarily diagnosed) to at least one of the environmental factors odorous/pungent chemicals, certain buildings, EMF, and everyday sounds [9].

Apart from general symptoms (e.g., fatigue and headache) that are common in these EI, certain symptoms seem to be more common in certain types of intolerance. For example, airway symptoms are common in intolerance to odorous/pungent chemicals [10], eye, upper respiratory and skin symptoms among nonspecific building-related symptoms [11], skin symptoms in intolerance attributed to EMF [12], and emotional symptoms and concentration difficulties in sound sensitivity [13]. Regarding EMF, skin symptoms dominate among those who attribute their symptoms to computer screens, fluorescent lamps and television sets, whereas those who attribute their symptoms to EMF in general have a more cognitive and emotional symptom picture [14,15]. The symptom picture varies within intolerances in general, and there is overlap between intolerances.

Studies of quality of life in EI have mostly been focused on individuals with severe MCS [16-20], with exception of sound sensitivity [21]. The impact on quality of life is predominantly manifested as not having access to society, and having difficulties keeping a job and maintaining social relations. Hence, in addition to health symptoms that per se are bothersome, attempts to avoid the symptoms by avoiding the environmental exposure results in isolation for the afflicted individual. Indeed, avoidance of the environmental exposure is the most commonly reported coping strategy in MCS [22], and is common also in symptom-attribution to EMF [23] and sounds [13].

Self-reports are important for diagnosing EI due to the lack of objective markers that are generally agreed on. In this context information on the afflicted individual’s symptomology is valuable, which also may contribute to the understanding of possible underlying mechanisms. For example, a symptom picture of predominantly airway symptoms may possibly indicate C-fiber hypersensitivity as in sensory hyperreactivity [24], whereas a picture of predominantly cognitive and affective symptoms may indicate an anxiety and stress-related condition [25].

Questionnaire instruments have been developed and metrically evaluated for assessment of specific symptoms in certain types of EI. These include the Quick Environmental Exposure and Sensitivity Inventory [26] and the Idiopathic Environmental Intolerance Symptom Inventory (IEISI) [10] for intolerance to odorous/pungent chemicals, and the MM-questionnaires for nonspecific building-related symptoms [27]. However, there is no documentation of metrically evaluated instruments for specific symptoms attributed to exposure to EMF or everyday sounds. Studies show that concomitant intolerance attributed to odorous/pungent chemicals, certain buildings, EMF, and sounds is common [9,12,28]. This motivates simultaneous investigation of these intolerances, which requests a questionnaire instrument that includes relevant symptoms in all these intolerances.

An objective of the current study was to develop and psychometrically evaluate a questionnaire instrument, referred to as the Environmental Hypersensitivity Symptom Inventory (EHSI), for asessment of symtomology in persons with symptoms attributed to odorous/pungent chemicals, certain buildings, EMF, and everyday sounds. The EHSI consists of 34 symptom items, requires limited time to respond to (about 5 min), yet it provides a detailed and broad description of the individual’s symptomology. The metric evaluation included investigation of dimensionality (factor structure) and reliability (internal consistency). The dimensionality was assessed with factor analysis to accomplish appropriate symptom categories. Thus, it is useful to group the symptoms into appropriate categories to enhance the responders’ evaluation of their symptom prevalence and the administrator’s interpretation of symptom pattern in the responses.

Another objective was to establish normative data for the EHSI. In addition to normative data for the general population, reference data were provided for combinations of specific age groups (young, middle-aged and elderly adults) and gender. These data referred to having had the specific symptom on a weekly basis over the preceding three months.

There can be a large difference in symptomology between individuals with EI. This suggests that a questionnaire-based instrument aimed at providing a detailed yet broad description of the individual’s symptom picture should provide the respondent with the possibility to also report symptoms that are not specifically listed in the questionnaire. The EHSI was therefore designed to also include open-ended questions about additional symptoms pertaining to certain symptom categories as well as to symptoms pertaining to additional, unspecified categories. The objectives of this study were addressed by means of data from a population-based study, the Västerbotten Environmental Health Study.

## Methods

### Population and sample

The Västerbotten Environmental Health Study is an embracing name for different investigations on the same general population regarding various forms of environmental hypersensitivity in Sweden. The study population, inhabitants in the county of Västerbotten in Northern Sweden, has an age and gender distribution that is very similar to that of Sweden in general [29]. A random sample, drawn from the municipal register, of 8600 individuals aged 18 to 79 years was invited to participate. The sample was stratified for age and gender according to the following age strata: 18–29, 30–39, 40–49, 50–59, 60–69, and 70–79 years. Of the 8600 individuals, 8520 could be reached, among whom 3406 (40.0%) agreed to participate. Age and gender distributions for the responders are given in Table 1. The sample is described in Table 2 with respect to demographics, smoking, and health conditions of relevance to symptomology in EI.

**Table 1:** Numbers of responders (and response percentages) across age and gender strata

**Age strata (years)**	**Women**	**Men**
18-29	307 (32.7%)	179 (17.7%)
30-39	266 (40.9%)	177 (25.2%)
40-49	288 (40.7%)	230 (31.3%)
50-59	367 (51.0%)	295 (39.7%)
60-69	405 (58.6%)	356 (50.7%)
70-79	265 (53.8%)	271 (63.9%)
18-79	1898 (45.2%)	1508 (34.9%)

**Table 2:** Sample characteristics (n = 3406)

	
Age, mean years (SD)	51.2 (16.8)
Women/men, n (%)	1898/1508 (55.7/44.3)
Education (highest), n (%)	
Primary school	823 (24.5)
High school	1137 (33.8)
University	1405 (41.8)
Smoker, n (%)	298 (8.8)
General health status, n (%)	
Very good or excellent	1349 (40.0)
Good	1152 (34.2)
Somewhat good or poor	868 (25.8)
Diagnosis^1^, n (%)	
Hypertension	838 (24.6)
Diabetes	186 (5.5)
Rheumatic disease	147 (4.3)
Disease in back, joints or muscles	492 (14.4)
Multiple chemical sensitivity	107 (3.1)
Nonspecific building-related symptoms	47 (1.4)
IEI-EMF^2^	15 (0.4)
Sound sensitivity	96 (2.8)
Asthma due to allergy	164 (4.8)
Asthma other than allergy	129 (3.8)
Allergic rhinitis	298 (8.7)
Atopic dermatatis	88 (2.6)
Migraine	151 (4.4)
Generaliserad anxiety disorder	32 (1.0)
Depression	170 (5.0)

^1^Self-report of having been given a diagnosis by a physician.

^2^Idiopathic environmental intolerance attributed to electromagnetic fields.

### The environmental hypersensitivity symptom inventory

The EHSI is to a large extent based on the IEISI that was developed for assessment of symptom prevalence attributed specifically to odorous/pungent chemicals [10]. The 27 specific symptoms in the IEISI were those reported by at least 20% of a sample with moderate to severe chemical intolerance. Many of these 27 symptoms are also commonly found in nonspecific building-related symptom [6,30], IEI-EMF [12,31,32], and sound sensitivity [13]. However, certain modifications and additions to the IEISI were made to better cover the symptomology of intolerance to certain buildings, EMF, and sounds. Thus, “skin irritation/redness” was replaced with the four items “facial itching/stinging/tightness/heat”, “facial redness”, “dry facial skin”, and “body itching”; and “head fullness/pressure” was replaced with the two items “head fullness” and “head pressure”. Furthermore, “nasal mucosa irritation/dryness”, “dry eyes”, and “general discomfort” were added. In total, the EHSI consists of 34 specific symptoms.

**Figure 1:**
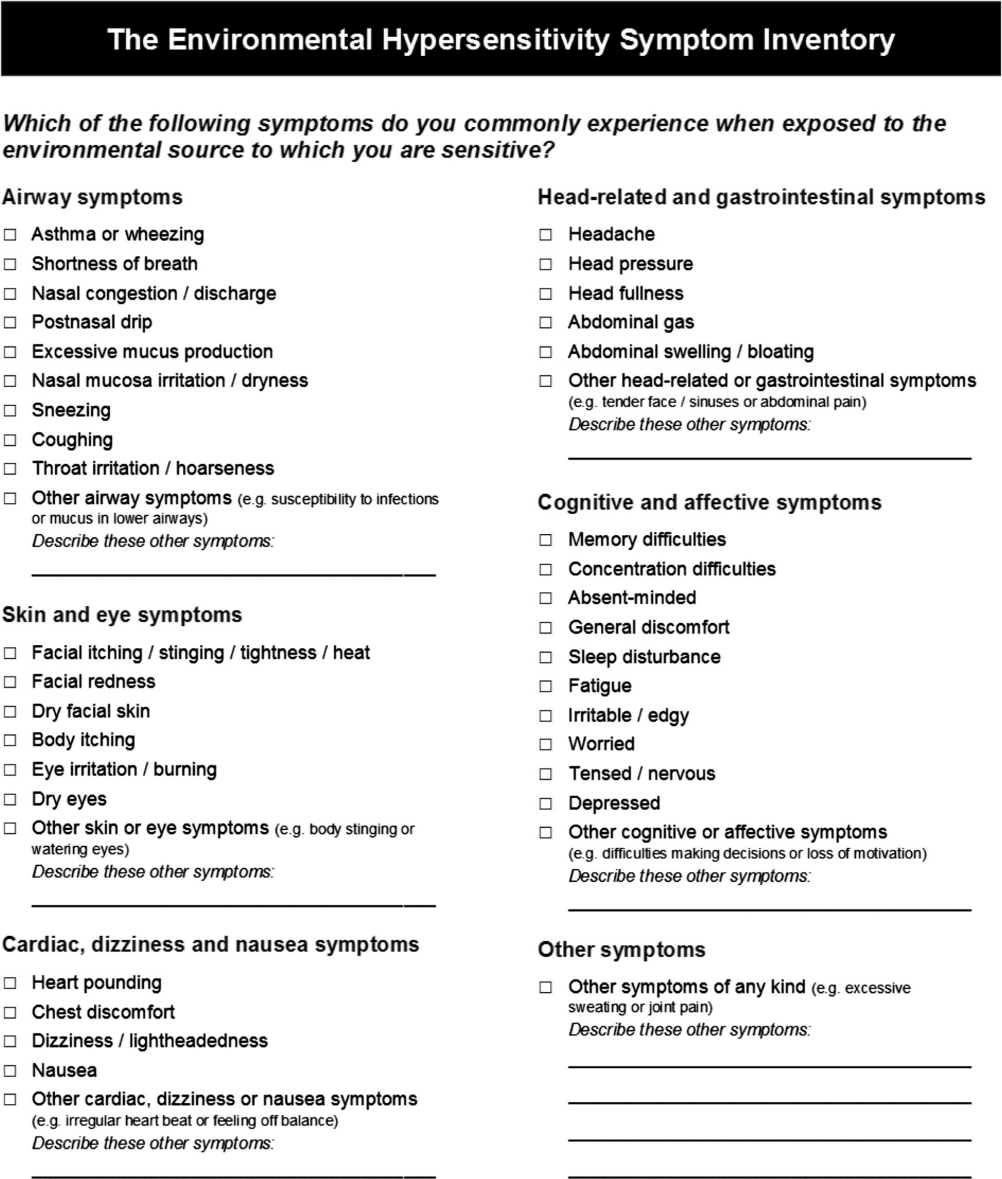
The environmental hypersensitivity symptom inventory.

Open-ended questions about additional symptoms pertaining to the symptom categories as well as to symptoms pertaining to additional, unspecified categories are also included in the EHSI. Since the likelihood of remembering to report a certain condition increases when that condition is provided to the respondent [33], examples of additional symptoms are given after each open-ended question in the EHSI. These examples were adopted from the IEISI. The final version of the EHSI is presented in Figure 1.

For the normative data, the frequency and time interval for a symptom to be considered as prevalent was having had the symptom every week over the preceding three months. This was partly based on the fact that this typically is used for the definition for nonspecific building-related symptoms [34], partly due to the three-month period being long enough to avoid memory effects and short enough to permit efficient follow-up studies after remedial measures have been taken [27].

### Procedure

A questionnaire was used that included the EHSI and questions regarding demographics, smoking, and health conditions (Table 2). The responders were mailed the questionnaire, to be returned by mail with prepaid postage. Non-responders received up to two reminders. All participants responded to the questionnaire during the period March-April, 2010, before the onset of the pollen season in Västerbotten. The study was conducted in accordance with the Helsinki Declaration and approved by the Umeå Regional Ethics Board. All responders gave their informed consent to participate.

### Statistical analysis

An exploratory factor analysis with Promax rotation and Kaiser normalization was conducted to study dimensionality of the 34 specific EHSI symptoms for categorization into symptom groups. An oblique factor rotation was chosen since prior studies of environmental hypersensitivity suggest strong commonalities among various types of somatic symptoms [4]. A scree test plot was made to identify the number of factors to be extracted [35]. The Kuder-Richardson Formula 20 coefficient (KR-20), comparable with the Cronbach alpha coefficient, was used for assessing internal consistency. Normative data for symptom prevalence were expressed in percentages among combinations of specific age groups [young (18–34 years), middle-aged (35–54 years), and elderly (55–79 years)] and gender, for the three age groups separately, for gender separately, and for the total sample. Since the response-rate in different age and gender strata varied, weighted prevalence rates for the entire sample were calculated as well. The weights used were calculated based on the inverse of the probability of respondents in each age and gender strata to participate [36].

## Results

### Dimensionality of the EHSI

**Figure 2:**
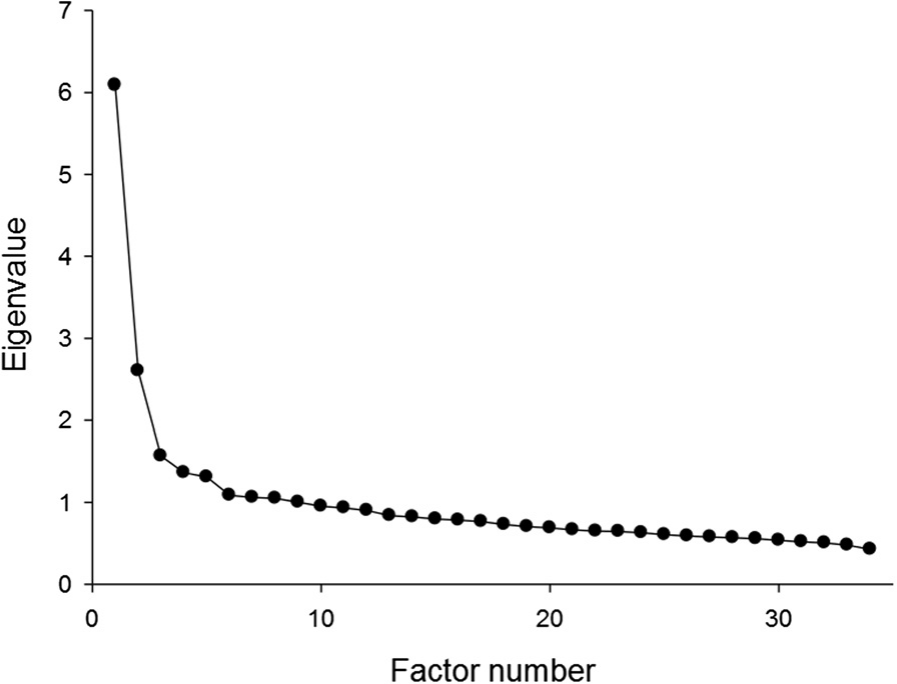
Scree test plot of eigenvalues for the 34 factors.

The factor analysis of the data for the 34 specific symptoms identified nine factors with an eigenvalue above 1. Their eigenvalues were 6.09 (17.90% explained variance), 2.60 (7.66%), 1.57 (4.61%), 1.36 (4.01%), 1.31 (3.86%), 1.09 (3.20%), 1.06 (3.12%), 1.05 (3.09%), and 1.01 (2.95%). However, a scree-test plot suggests only five factors to be extracted (Figure 2; the number of factors preceding the last “elbow”; [35]). The factor loadings of each EHSI item on each of the five factors are presented in Table 3.

**Table 3:** Factor loadings with the strongest loading for each symptom item given in bold

	**1**	**2**	**3**	**4**	**5**
Concentration difficulties	**.695**	.145	.273	.216	.260
Depressed	**.667**	.084	.149	.235	.248
Worried	**.649**	.072	.154	.298	.239
Tensed/nervous	**.644**	.046	.189	.317	.210
Absent-minded	**.613**	.159	.210	.107	.223
General discomfort	**.607**	.080	.152	.428	.182
Irritable/edgy	**.599**	.090	.170	.174	.322
Memory difficulties	**.564**	.205	.263	.172	.142
Fatigue	**.545**	.142	.243	.102	.466
Sleep disturbance	**.402**	.163	.209	.101	.299
Coughing	.119	**.683**	.106	.240	.072
Throat irritation/hoarseness	.119	**.665**	.176	.239	.122
Shortness of breath	.174	**.656**	.124	.528	-.131
Excessive mucus production	.141	**.587**	.196	.246	-.057
Postnasal drip	.128	**.564**	.273	.106	.137
Nasal congestion/discharge	.133	**.555**	.194	.059	.327
Sneezing	.139	**.555**	.175	.064	.349
Irritation/dryness of the nasal mucosa	.145	**.497**	.408	.061	.236
Asthma or wheezing	.044	**.476**	.093	.397	-.253
Facial itching/stinging/tightness/heat	.205	.184	**.691**	.201	.076
Facial redness	.221	.136	**.689**	.134	.074
Dry facial skin	.259	.133	**.599**	.057	.266
Dry eyes	.090	.238	**.509**	.169	.213
Body itching	.207	.197	**.478**	.154	.147
Eye irritation/burning	.192	.350	**.460**	.173	.247
Chest discomfort	.238	.188	.143	**.602**	.106
Heart pounding	.264	.126	.206	**.567**	.136
Nausea	.270	.167	.107	**.493**	.424
Dizziness/lightheadedness	.238	.159	.254	**.476**	.313
Abdominal gas	.217	.167	.216	.040	**.581**
Abdominal swelling/bloating	.267	.114	.220	.195	**.562**
Headache	.273	.129	.069	.173	**.535**
Head fullness	.359	.187	.215	.307	**.438**
Head pressure	.281	.078	.162	.367	**.399**

The ten items that loaded strongest on Factor 1 can be referred to as cognitive and affective symptoms; the nine items that loaded strongest on Factor 2 can be referred to as airway symptoms; the six items that loaded strongest on Factor 3 can be referred to as skin and eye symptoms; the four items that loaded strongest on Factor 4 can be referred to as cardiac, dizziness and nausea symptoms; and the five items that loaded strongest on Factor 5 can be referred to as head-related and gastrointestinal symptoms.

### Reliability and normative data of the EHSI

The KR-20 coefficient was 0.74 for airway symptoms, 0.60 for skin and eye symptoms, 0.55 for cardiac, dizziness and nausea symptoms, 0.55 for head-related and gastrointestinal symptoms, 0.80 for cognitive and affective symptoms, and 0.85 for the entire 34-item EHSI. Normative data are given in Table 4 for prevalence of specific symptoms expressed in percentages of subpopulations who report having each symptom every week over the preceding three months.

**Table 4:** Percentage reporting having had symptoms every week over the preceding three months, constituting normative data

			**Middle-**	**Middle-**				**All**				**Total**	**Total**
	**Young**	**Young**	**aged**	**aged**	**Elderly**	**Elderly**	**All**	**middle-**	**All**	**All**	**All**	**sample**	**sample**
	**women**	**men**	**women**	**men**	**women**	**men**	**young**	**aged**	**elderly**	**women**	**men**	**unweighted**	**weighted**
	**(n = 441)**	**(n = 265)**	**(n = 597)**	**(n = 455)**	**(n = 860)**	**(n = 788)**	**(n = 706)**	**(n = 1052)**	**(n = 1648)**	**(n = 1898)**	**(n = 1508)**	**(n = 3406)**	**(n = 3406)**
**Airway symptoms**													
Asthma or wheezing	5.0	6.0	6.9	7.7	11.0	11.4	5.4	7.2	11.2	8.3	9.4	8.8	8.1
Shortness of breath	7.7	6.4	8.2	7.5	11.6	12.2	7.2	7.9	11.9	9.6	9.7	9.7	9.0
Nasal congestion/discharge	28.1	21.9	25.0	21.8	25.1	29.2	25.8	23.6	27.1	25.8	25.7	25.7	25.0
Postnasal drip	8.4	6.8	9.0	6.8	12.2	9.4	7.8	8.1	10.8	10.3	8.2	9.3	8.7
Excessive mucus production	3.4	3.8	4.2	5.7	8.1	8.8	3.5	4.8	8.4	5.8	7.0	6.3	5.7
Nasal mucosa irritation/dryness	13.8	8.7	22.8	12.5	28.8	17.8	11.9	18.3	23.5	23.4	14.6	19.5	17.3
Sneezing	27.9	20.4	24.1	23.7	30.8	26.8	25.1	24.0	28.9	28.0	24.7	26.6	25.6
Coughing	15.4	12.5	17.3	16.0	23.3	20.8	14.3	16.7	22.1	19.5	17.9	18.8	17.4
Throat irritation/hoarseness	11.6	9.8	12.7	10.8	19.8	17.1	10.9	11.9	18.5	15.6	13.9	14.9	13.8
**Skin and eye symptoms**													
Facial itching/stinging/tightness/heat	5.9	0.4	6.5	3.3	7.2	3.8	3.8	5.1	5.6	6.7	3.1	5.1	4.6
Facial redness	7.0	3.0	8.2	4.2	7.2	4.1	5.5	6.5	5.7	7.5	3.9	5.9	5.5
Dry facial skin	37.2	15.1	24.3	13.4	20.0	8.5	28.9	19.6	14.5	25.3	11.1	19.1	19.1
Body itching	14.3	5.7	14.1	13.2	14.5	12.4	11.0	13.7	13.5	14.3	11.5	13.1	12.2
Eye irritation/burning	8.6	7.5	14.4	9.9	18.8	13.5	8.2	12.5	16.3	15.1	11.3	13.4	12.1
Dry eyes	11.8	7.2	14.7	8.8	21.6	11.3	10.1	12.2	16.7	17.2	9.8	13.9	12.5
**Cardiac, dizziness and nausea symptoms**													
Heart pounding	8.8	1.9	8.9	6.4	12.1	7.0	6.2	7.8	9.6	10.3	5.9	8.4	7.5
Chest discomfort	6.8	3.4	5.4	5.3	7.6	7.4	5.5	5.3	7.5	6.7	6.0	6.4	6.0
Dizziness/lightheadedness	10.9	1.5	8.9	3.3	10.6	7.6	7.4	6.5	9.2	10.1	5.2	8.0	6.9
Nausea	14.3	2.3	7.2	5.5	5.8	2.8	9.8	6.5	4.4	8.2	3.5	6.1	6.1
**Head-related and gastrointestinal symptoms**													
Headache	41.0	23.8	36.3	24.0	23.1	9.9	34.6	31.0	16.8	31.5	16.6	24.9	25.9
Head pressure	10.7	3.8	9.5	5.5	4.9	2.7	8.1	7.8	3.8	7.7	3.7	5.9	6.0
Head fullness	19.0	9.8	15.1	10.5	11.9	6.0	15.6	13.1	9.0	14.5	8.0	11.7	11.9
Abdominal gas	44.9	32.5	37.7	37.1	37.0	30.8	40.2	37.5	34.0	39.0	33.0	36.4	36.5
Abdominal swelling/bloating	29.7	7.2	21.9	11.2	17.1	8.8	21.2	17.3	13.1	21.5	9.2	16.1	15.8
**Cognitive and affective symptoms**													
Memory difficulties	13.4	9.8	20.3	14.1	14.8	17.1	12.0	17.6	15.9	16.2	14.9	15.6	14.7
Concentration difficulties	24.9	16.6	22.3	16.0	13.7	8.8	21.8	19.6	11.3	19.0	12.3	16.1	16.7
Absent-minded	33.8	23.8	25.3	15.8	15.5	12.2	30.0	21.2	13.9	22.8	15.3	19.5	20.7
General discomfort	12.2	3.8	8.5	7.5	4.0	2.7	9.1	8.1	3.3	7.3	4.3	6.0	6.3
Sleep disturbance	26.1	16.6	32.3	22.6	33.6	19.5	22.5	28.1	26.9	31.5	20.0	26.4	24.9
Fatigue	62.8	41.1	55.6	39.6	38.6	25.8	54.7	48.7	32.5	49.6	32.6	42.1	43.2
Irritable/edgy	31.7	21.5	26.3	17.1	10.3	7.7	27.9	22.3	9.1	20.3	13.0	17.1	18.6
Worried	29.3	18.1	21.4	18.0	17.4	9.1	25.1	20.0	13.5	21.4	13.4	17.9	18.6
Tensed/nervous	23.4	8.3	16.2	10.8	7.0	2.7	17.7	13.9	4.9	13.7	6.1	10.3	11.1
Depressed	31.5	16.2	18.1	15.2	14.8	8.0	25.8	16.8	11.5	19.7	11.6	16.1	17.0

Young = 18–34 years, middle-aged = 35–54 years, elderly = 55–79 years.

## Discussion

An objective of the present study was to develop and psychometrically evaluate a questionnaire-based instrument, the EHSI, for assessment of symptom prevalence in persons with common types of environmental hypersensitivity. The instrument was aimed at requiring limited time to respond to, yet provide a detailed and broad description of the symptomology of the individual or group under study. Although the symtomology was focused on persons who attribute their symptoms to odorous/pungent chemicals, certain buildings, EMF, and sounds, the wide range of symptoms in the EHSI is likely to cover the symtomology of several other types of environmental hypersensitivity, including asthma and allergy.

The evaluation of the EHSI suggests a factor structure of five factors: airway symptoms, skin and eye symptoms, cardiac, dizziness and nausea symptoms, head-related and gastrointestinal symptoms, and cognitive and affective symptoms. The grouping of the cardiac, dizziness and nausea symptoms can be explained by sympathetic activity such that extensive heart pounding can cause chest discomfort, nausea and dizziness. Grouping of head-related and gastrointestinal symptoms can be referred to both types of symptoms being common psychosomatic symptoms. As would be expected, the head-related and gastrointestinal symptoms were found to load relatively high on the cognitive and affective factor. The outcome from the factor analysis was similar to that reported by Miller and Mitzel [37] and by Andersson and associates [10], also using factor analysis, for those symptoms that were in common between studies.

The internal consistency of the entire EHSI and the symptom category cognitive and affective symptoms can be considered as good, the symptom category airway symptoms can be considered as acceptable, the symptom category skin and eye symptoms can be considered as questionable, and the symptom categories cardiac, dizziness and nausea symptoms, and head-related and gastrointestinal symptoms can be considered as poor. The poor consistency in the latter two symptom categories is likely to be due to these categories having few items in combination with being rather broad. A consequence is that use of a composite measure of cardiac, dizziness and nausea symptoms, and head-related and gastrointestinal symptoms from the EHSI should be supplemented with inspection of whether there is a large variability between the symptoms in this category. The validity of the EHSI was not investigated in this study. One reason for this is that the majority of its symptoms have been validated in a prior study of environmental hypersensitivity [10]. Another reason is the simplicity of assessment with the EHSI: having a specific symptom or not. Thus, the face validity [38] of the EHSI can be considered as good.

Another objective of the study was to provide normative data for various subgroups of age and gender, and for the general adult population. The population-based nature of the Västerbotten Environmental Health Study and the fact that the study population has an age and gender distribution that is very similar to that of Sweden in general [29] enhances the representativeness. However, among the randomly selected individuals only 40% volunteered, which compromises the representativeness. Research ethical regulations for conducting research in Sweden do not allow asking the selected individuals why they chose not to participate or about certain characteristics they may possess [39]. However, information on age and gender was available for those who declined participation in this study, and the largest proportion of non-responders was found among young men (Table 1). The generally low response rate increases the risk of a selection bias. Thus, the special topic of the study (environmental health) may have attracted, in particular, respondents with health problems attributed to environmental aspects [40], which may have resulted in the prevalence rates being higher than otherwise would have been the case. Comparisons with data from prior Swedish population-based studies do only partly support the notion that the current prevalence rates are too high. Whereas Eriksson and Stenberg [34] reported prevalence rates for adults aged 18–64 years that were generally lower than in the present study, Andersson and Norlén [41] reported rates based on all ages that were generally higher. The generally higher prevalence rates in women than in men (Table 4) accord with typical results on gender differences [42], and the pattern of age-related differences corresponds in general with prior Swedish population-based data for young and middle-aged adults [34].

The applicability of the EHSI is not limited to assessment of having had the specific symptoms every week over the preceding three months, or to assessment of prevalence (yes/no), for which the normative data are valid. The instrument can also be used for assessing the prevalence of symptoms as a direct result of the environmental exposure. Furthermore, the respondent can rate to what extent he/she experiences each symptom. An example of an appropriate rating scale for such a purpose is the Environmental Annoyance Scale, which is a category scale with seven semantic descriptors [Not at all (0), a little (1), partly (2), pretty much (3), rather much (4), to a large extent (5), and extremely much (6)], and with ratio-scale properties and good reliability and validity [43].

## Conclusions

The 34-item EHSI for assessment of symptoms in various types of environmental hypersensitivity requires limited time to respond to, yet provides a detailed and broad description of the symptomology, including airway, skin, eye, cardiac, dizziness, nausea, head-related, gastrointestinal, cognitive and affective symptoms. Measures of internal consistency suggest that symptom prevalence can reliably be combined for a composite measure for the entire EHSI and for the symptom categories airway symptoms, skin and eye symptoms, and cognitive and affective symptoms. In contrast, caution should be taken when combining items for the symptom categories cardiac, dizziness and nausea symptoms, and head-related and gastrointestinal symptoms. Normative data for various subgroups of age and gender, and for the general adult population are available for having had the specific symptoms every week over the preceding three months.

## Competing interests

The authors declare that they have no competing interests.

## Authors’ contributions

All authors contributed in planning the study. SN and EP organized the data collection. SN drafted the article, and EP, ASC and BS read and approved the final version of the manuscript.
